# An algorithm for the split-feasibility problems with application to the split-equality problem

**DOI:** 10.1186/s13660-017-1567-9

**Published:** 2017-12-08

**Authors:** Chih-Sheng Chuang, Chi-Ming Chen

**Affiliations:** 1 Department of Applied Mathematics, National Chiayi University, Chiayi, Taiwan; 2 Institute of Computational and Modeling Science, National Tsing Hua University, Hsinchu, Taiwan

**Keywords:** 49J53, 49M37, 90C25, split-feasibility problem, split-equality problem, linear inverse problem, projection

## Abstract

In this paper, we study the split-feasibility problem in Hilbert spaces by using the projected reflected gradient algorithm. As applications, we study the convex linear inverse problem and the split-equality problem in Hilbert spaces, and we give new algorithms for these problems. Finally, numerical results are given for our main results.

## Introduction

The split-feasibility problem was first introduced by Censor *et al.* [1]:

$$ (\mathbf{SFP}) \quad\text{Find } \bar{x}\in H_{1} \text{ such that }\bar{x}\in C \text{ and }A\bar{x}\in Q, $$

where *C* is a nonempty closed convex subset of a real Hilbert space $H_{1}$, *Q* is a nonempty closed convex subset of a real Hilbert space $H_{2}$, and $A:H_{1}\rightarrow H_{2}$ is a linear and bounded operator. The split-feasibility problem was originally introduced by Censor and Elfving [2] for modeling phase retrieval problems, and it later was studied extensively as an extremely powerful tool for the treatment of a wide range of inverse problems, such as medical image reconstruction and intensity-modulated radiation therapy problems. For examples, one may refer to [2–4].

In 2002, Byrne [5] proposed the CQ algorithm to study the split-feasibility problem:

1.1 $$ \text{(CQ algorithm)}\quad \textstyle\begin{cases} x_{1}\text{ is chosen arbitrarily in }\mathbb{R}^{n},\\ x_{n+1}=P_{C} (x_{n}-\rho_{n} A^{\top}(I-P_{Q})Ax_{n}), \quad n\in\mathbb{N}, \end{cases} $$

where *C* is a nonempty closed convex subset of $\mathbb{R}^{\ell}$, *Q* is a nonempty closed convex subset of $\mathbb{R}^{m}$, $\{\rho_{n}\} _{n\in\mathbb{N}}$ is a sequence in the interval $(0,2/ \Vert A \Vert ^{2})$, $P_{C}$ is the metric projection from $\mathbb{R}^{\ell}$ onto *C*, $P_{Q}$ is the metric projection from $\mathbb{R}^{m}$ onto *Q*, *A* is an $m\times\ell$ matrix, and $A^{\top}$ is the transpose of *A*.

In 2005, Qu and Xiu [6] presented modifications of the CQ algorithm in the setting of finite dimensional spaces by adopting the Armijo-like searches, which need not compute the matrix inverses and the largest eigenvalue of the matrix $A^{\top}A$. In 2007, Censor, Motova, and Segal [4] studied the multiple-sets split-feasibility problem that requires one to find a point closest to a family of closed convex sets in one space such that its image under a linear transformation will be closest to another family of closed convex sets in the image space by using a perturbed projection method.

In 2010, Xu [7] gave the following modified CQ algorithm and gave a weak convergence theorem for the split-feasibility problem in infinite dimensional Hilbert spaces:

1.2 $$ \textstyle\begin{cases} x_{1}\text{ is chosen arbitrarily in }\mathbb{R}^{n},\\ x_{n+1}:=P_{C}(x_{n}-\rho_{n}(A^{*}(I-P_{Q})Ax_{n})),\quad n\in\mathbb{N}, \end{cases} $$

where $\{\rho_{n}\}_{n\in\mathbb{N}}$ is chosen in the interval $(0,2/ \Vert A \Vert ^{2})$, *C* is a nonempty closed convex subset of a real Hilbert space $H_{1}$, *Q* is a nonempty closed convex subset of a real Hilbert space $H_{2}$, and $A:H_{1}\rightarrow H_{2}$ is a linear and bounded operator, and let $A^{*}$ be the adjoint of *A*.

Besides, Xu [7] also gave a regularized algorithm for the split-feasibility problem and proposed a strong convergence theorem under suitable conditions:

1.3 $$ \textstyle\begin{cases} x_{1}\text{ is chosen arbitrarily in }\mathbb{R}^{n},\\ x_{n+1}:=P_{C}((1-a_{n}\rho_{n})x_{n}-\rho_{n}(A^{*}(I-P_{Q})Ax_{n})),\quad n\in\mathbb{N}, \end{cases} $$

where *C* is a nonempty closed convex subset of a real Hilbert space $H_{1}$, *Q* is a nonempty closed convex subset of a real Hilbert space $H_{2}$, and $A:H_{1}\rightarrow H_{2}$ is a linear and bounded operator, and $A^{*}$ is the adjoint of *A*.

In 2015, Qu, Liu, and Zheng [8] gave the following modified CQ algorithm to study the split-feasibility problem:

$$ \text{(CQ-like algorithm)}\quad \textstyle\begin{cases} x_{1}\text{ is chosen arbitrarily in }H_{1},\\ x_{n+1}=P_{C} (x_{n}+w_{n}r_{n} A^{\top}(P_{Q}-I)Ax_{n}), \end{cases} $$

where $0<\underline{w}\leq w_{n}\leq\overline{w}<2$, and $r_{n}=\frac { \Vert (P_{Q}-I)Ax_{n} \Vert ^{2}}{ \Vert A^{\top}(P_{Q}-I)Ax_{n} \Vert ^{2}}$. Indeed, Qu *et al.* [8] thought that the CQ-like algorithm not only need not compute the largest eigenvalue of the related matrix but also need not use any line search scheme.

For more details as regards various algorithms for the split-feasibility problems and related problems, one may refer to [5–20] and related references.

Motivated by the above work, in this paper, we study the split-feasibility problem in Hilbert spaces by using the projected reflected gradient algorithm. As applications, we study the convex linear inverse problem and the split-equality problem in Hilbert spaces, and give new algorithms for these problems. Final, numerical results are given for our main results.

## Preliminaries

Let *H* be a real Hilbert space with inner product $\langle\cdot ,\cdot\rangle$ and norm $\Vert \cdot \Vert $. We denote the strong convergence and weak convergence $\{x_{n}\}_{n\in\mathbb{N}}$ to $x\in H$ by $x_{n}\rightarrow x$ and $x_{n}\rightharpoonup x$, respectively. From [21], for each $x,y,u,v\in H$ and $\lambda\in\mathbb{R}$, we have

2.1 $$\begin{aligned} & \Vert x+y \Vert ^{2}= \Vert x \Vert ^{2}+2\langle x,y\rangle+ \Vert y \Vert ^{2}, \end{aligned}$$

2.2 $$\begin{aligned} & \bigl\Vert \lambda x+(1-\lambda)y \bigr\Vert ^{2}= \lambda \Vert x \Vert ^{2}+(1-\lambda ) \Vert y \Vert ^{2}-\lambda(1-\lambda) \Vert x-y \Vert ^{2}, \end{aligned}$$

2.3 $$\begin{aligned} &2\langle x-y,u-v\rangle= \Vert x-v \Vert ^{2}+ \Vert y-u \Vert ^{2}- \Vert x-u \Vert ^{2}- \Vert y-v \Vert ^{2}. \end{aligned}$$

### Definition 2.1

Let *C* be a nonempty closed convex subset of a real Hilbert space *H*, and $T:C\rightarrow H$ be a mapping, and set $\operatorname{Fix}(T):=\{x\in C: Tx=x\}$. Thus,

(i) *T* is a nonexpansive mapping if $\Vert Tx-Ty \Vert \leq \Vert x-y \Vert $ for every $x,y\in C$.
(ii) *T* is a firmly nonexpansive mapping if $\Vert Tx-Ty \Vert ^{2}\leq \langle x-y,Tx-Ty\rangle$ for every $x,y\in C$, that is, $\Vert Tx-Ty \Vert ^{2}\leq \Vert x-y \Vert ^{2}- \Vert (I-T)x-(I-T)y \Vert ^{2}$ for every $x,y\in C$.
(iii) *T* is a quasi-nonexpansive mapping if $\operatorname{Fix}(T)\neq \emptyset$ and $\Vert Tx-y \Vert \leq \Vert x-y \Vert $ for every $x\in C$ and $y\in \operatorname{Fix}(T)$.

### Remark 2.1

If *T* is a firmly nonexpansive mapping, then *T* is a nonexpansive mapping.

### Lemma 2.1

([22])

*Let* *C* *be a nonempty closed convex subset of a real Hilbert space* *H*. *Let* $T:C\rightarrow H$ *be a nonexpansive mapping*, *and* $\{x_{n}\}_{n\in\mathbb{N}}$ *be a sequence in* *C*. *If* $x_{n}\rightharpoonup w$ *and* $\lim_{n\rightarrow \infty} \Vert x_{n}-Tx_{n} \Vert =0$, *then* $Tw=w$.

Let *C* be a nonempty closed convex subset of a real Hilbert space *H*. For each $x\in H$, there is a unique element $\bar{x}\in C$ such that

$$\Vert x-\bar{x} \Vert =\min_{y\in C} \Vert x-y \Vert . $$

In this study, we set $P_{C}x=\bar{x}$, and $P_{C}$ is called the metric projection from *H* onto *C*.

### Lemma 2.2

([21])

*Let* *C* *be a nonempty closed convex subset of a real Hilbert space* *H*, *and let* $P_{C}$ *be the metric projection from* *H* *onto* *C*. *Then the following are satisfied*:

(i) $\langle x-P_{C}x,P_{C}x-y\rangle\geq0$ *for all* $x\in H$ *and* $y\in C$;
(ii) $\Vert x-P_{C}x \Vert ^{2}+ \Vert P_{C}x-y \Vert ^{2}\leq \Vert x-y \Vert ^{2}$ *for all* $x\in H$ *and* $y\in C$;
(iii) $P_{C}$ *is a firmly nonexpansive mapping*.

### Lemma 2.3

([23])

*Let* $H_{1}$ *and* $H_{2}$ *be two real Hilbert spaces*, $A:H_{1}\rightarrow H_{2}$ *be a linear mapping*, *and* $A^{*}$ *be the adjoint of* *A*. *Let* *C* *be a nonempty closed convex subset of* $H_{2}$. *Let* $T:=A^{*}(I-P_{C})A$. *Then* *T* *is a monotone mapping*. *In fact*, *we have*

$$ \bigl\Vert (I-P_{Q})Ax-(I-P_{Q})Ay \bigr\Vert ^{2}\leq\bigl\langle x-y,A^{*}(I-P_{Q})Ax-A^{*}(I-P_{Q})Ay \bigr\rangle $$

*for all* $x,y\in H_{1}$.

## Projected reflected gradient algorithm

### Theorem 3.1

*Let* $H_{1}$ *and* $H_{2}$ *be real Hilbert spaces*, *C* *and* *Q* *be nonempty closed convex subsets of* $H_{1}$ *and* $H_{2}$, *respectively*, *and* $A:H_{1}\rightarrow H_{2}$ *be a linear and bounded operator with adjoint operator* $A^{*}$. *Let* Ω *be the solution set of the split*-*feasibility problem and assume that* $\Omega\neq\emptyset$. *For* $k>0$, *suppose* *ρ* *satisfies*

$$0< \rho< \min \biggl\{ \frac{\sqrt{k}}{(1+\sqrt{k})\cdot \Vert A \Vert ^{2}},\frac{k}{(k\sqrt{k}+\sqrt{k}+1)\cdot \Vert A \Vert ^{2}} \biggr\} . $$

*Let* $\{x_{n}\}_{n\in\mathbb{N}}$ *be defined by*

3.1 $$ \textstyle\begin{cases} x_{1}\textit{ is chosen arbitrarily in }H_{1},\\ y_{1}=x_{1},\\ x_{n+1}:=P_{C}(x_{n}-\rho A^{*}(I-P_{Q})Ay_{n}),\\ y_{n+1}:=2x_{n+1}-x_{n},\quad n\in\mathbb{N}. \end{cases} $$

*Then there exists* $\bar{x}\in\Omega$ *such that* $\{x_{n}\}_{n\in \mathbb{N}}$ *converges weakly to* *x̄*.

### Proof

Let $v\in C$, $w\in\Omega$, and $n\in\mathbb{N}$ be fixed. Then, by Lemma 2.2, we have

3.2 $$\begin{aligned} & \Vert x_{n+1}-v \Vert ^{2} \\ &\quad= \bigl\Vert P_{C}\bigl(x_{n}-\rho A^{*}(I-P_{Q})Ay_{n} \bigr)-P_{C} v \bigr\Vert ^{2} \\ &\quad\leq \bigl\Vert x_{n}-\rho A^{*}(I-P_{Q})Ay_{n})-v \bigr\Vert ^{2}- \bigl\Vert x_{n+1}-x_{n}+\rho A^{*}(I-P_{Q})Ay_{n} \bigr\Vert ^{2} \\ &\quad= \Vert x_{n}-v \Vert ^{2}+\rho^{2} \bigl\Vert A^{*}(I-P_{Q})Ay_{n} \bigr\Vert ^{2}-2\rho \bigl\langle x_{n}-v,A^{*}(I-P_{Q})Ay_{n}\bigr\rangle \\ &\qquad{}- \Vert x_{n+1}-x_{n} \Vert ^{2}- \rho^{2} \bigl\Vert A^{*}(I-P_{Q})Ay_{n} \bigr\Vert ^{2}-2\rho\bigl\langle x_{n+1}-x_{n},A^{*}(I-P_{Q})Ay_{n} \bigr\rangle \\ &\quad= \Vert x_{n}-v \Vert ^{2}- \Vert x_{n+1}-x_{n} \Vert ^{2}-2\rho\bigl\langle x_{n+1}-v,A^{*}(I-P_{Q})Ay_{n}\bigr\rangle . \end{aligned}$$

By Lemma 2.3, we know that

3.3 $$ \bigl\langle y_{n}-v,A^{*}(I-P_{Q})Ay_{n}-A^{*}(I-P_{Q})Av \bigr\rangle \geq \bigl\Vert (I-P_{Q})Ay_{n}-(I-P_{Q})Av \bigr\Vert ^{2}. $$

Then, by (3.2) and (3.3),

3.4 $$\begin{aligned} & \Vert x_{n+1}-v \Vert ^{2}+2 \rho \bigl\Vert (I-P_{Q})Ay_{n}-(I-P_{Q})Av \bigr\Vert ^{2} \\ &\quad\leq \Vert x_{n}-v \Vert ^{2}- \Vert x_{n+1}-x_{n} \Vert ^{2}-2\rho\bigl\langle x_{n+1}-v,A^{*}(I-P_{Q})Ay_{n}\bigr\rangle \\ &\qquad{}+2\rho\bigl\langle y_{n}-v,A^{*}(I-P_{Q})Ay_{n}-A^{*}(I-P_{Q})Av \bigr\rangle \\ &\quad= \Vert x_{n}-v \Vert ^{2}- \Vert x_{n+1}-x_{n} \Vert ^{2}-2\rho\bigl\langle x_{n+1}-y_{n},A^{*}(I-P_{Q})Ay_{n}\bigr\rangle \\ &\qquad{}-2\rho\bigl\langle y_{n}-v,A^{*}(I-P_{Q})Av\bigr\rangle \\ &\quad= \Vert x_{n}-v \Vert ^{2}- \Vert x_{n+1}-x_{n} \Vert ^{2} \\ &\qquad{}-2\rho\bigl\langle x_{n+1}-y_{n},A^{*}(I-P_{Q})Ay_{n}-A^{*}(I-P_{Q})Ay_{n-1} \bigr\rangle \\ &\qquad{}-2\rho\bigl\langle x_{n+1}-y_{n},A^{*}(I-P_{Q})Ay_{n-1} \bigr\rangle -2\rho\bigl\langle y_{n}-v,A^{*}(I-P_{Q})Av\bigr\rangle . \end{aligned}$$

By Lemma 2.2, we know that

3.5 $$ \bigl\langle x_{n-1}-\rho A^{*}(I-P_{Q})Ay_{n-1}-x_{n},x_{n}-v \bigr\rangle \geq0, $$

and this implies that

3.6 $$ \textstyle\begin{cases} \langle x_{n-1}-\rho A^{*}(I-P_{Q})Ay_{n-1}-x_{n},x_{n}-x_{n+1}\rangle\geq 0,\\ \langle x_{n-1}-\rho A^{*}(I-P_{Q})Ay_{n-1}-x_{n},x_{n}-x_{n-1}\rangle\geq0. \end{cases} $$

Therefore, by (3.6),

3.7 $$ \bigl\langle x_{n-1}-\rho A^{*}(I-P_{Q})Ay_{n-1}-x_{n},2x_{n}-x_{n-1}-x_{n+1} \bigr\rangle \geq0. $$

That is,

3.8 $$ \bigl\langle x_{n-1}-\rho A^{*}(I-P_{Q})Ay_{n-1}-x_{n},y_{n}-x_{n+1} \bigr\rangle \geq0. $$

This implies that

3.9 $$\begin{aligned} &2\rho\bigl\langle A^{*}(I-P_{Q})Ay_{n-1},y_{n}-x_{n+1} \bigr\rangle \\ &\quad \leq 2\langle x_{n-1}-x_{n},y_{n}-x_{n+1} \rangle \\ &\quad=2\langle x_{n}-y_{n},y_{n}-x_{n+1} \rangle \\ &\quad= \Vert x_{n}-x_{n+1} \Vert ^{2}- \Vert x_{n}-y_{n} \Vert ^{2}- \Vert x_{n+1}-y_{n} \Vert ^{2}. \end{aligned}$$

Also, we have

3.10 $$\begin{aligned} &2\rho\bigl\langle y_{n}-x_{n+1},A^{*}(I-P_{Q})Ay_{n}-A^{*}(I-P_{Q})Ay_{n-1} \bigr\rangle \\ &\quad \leq 2\rho \Vert A \Vert ^{2}\cdot \Vert y_{n}-x_{n+1} \Vert \cdot \Vert y_{n}-y_{n-1} \Vert \\ &\quad\leq\rho \Vert A \Vert ^{2}\cdot\biggl(\sqrt{k} \Vert y_{n}-x_{n+1} \Vert ^{2}+\frac { \Vert y_{n}-y_{n-1} \Vert ^{2}}{\sqrt{k}}\biggr) \\ &\quad \leq\sqrt{k}\rho \Vert A \Vert ^{2}\cdot \Vert y_{n}-x_{n+1} \Vert ^{2}+\frac{\rho \Vert A \Vert ^{2}}{\sqrt{k}}\cdot \Vert y_{n}-y_{n-1} \Vert ^{2} \\ &\quad\leq\sqrt{k}\rho \Vert A \Vert ^{2}\cdot \Vert y_{n}-x_{n+1} \Vert ^{2}+\frac{\rho \Vert A \Vert ^{2}}{\sqrt{k}}\cdot \bigl( \Vert y_{n}-x_{n} \Vert + \Vert x_{n}-y_{n-1} \Vert \bigr)^{2} \\ &\quad= \sqrt{k}\rho \Vert A \Vert ^{2}\cdot \Vert y_{n}-x_{n+1} \Vert ^{2} \\ &\qquad{}+\frac{\rho \Vert A \Vert ^{2}}{\sqrt{k}}\cdot \bigl( \Vert y_{n}-x_{n} \Vert ^{2}+ \Vert x_{n}-y_{n-1} \Vert ^{2}+2 \Vert y_{n}-x_{n} \Vert \cdot \Vert x_{n}-y_{n-1} \Vert \bigr) \\ &\quad\leq\sqrt{k}\rho \Vert A \Vert ^{2}\cdot \Vert y_{n}-x_{n+1} \Vert ^{2} \\ &\qquad{}+\frac{\rho \Vert A \Vert ^{2}}{\sqrt{k}}\cdot \biggl( \Vert y_{n}-x_{n} \Vert ^{2}+ \Vert x_{n}-y_{n-1} \Vert ^{2}+\sqrt{k} \Vert y_{n}-x_{n} \Vert ^{2}+\frac {1}{\sqrt{k}} \Vert x_{n}-y_{n-1} \Vert \biggr) \\ &\quad = \sqrt{k}\rho \Vert A \Vert ^{2}\cdot \Vert y_{n}-x_{n+1} \Vert ^{2}+\rho \Vert A \Vert ^{2}\cdot\frac {1+\sqrt{k}}{\sqrt{k}} \Vert y_{n}-x_{n} \Vert ^{2} \\ &\qquad{}+\rho \Vert A \Vert ^{2}\cdot \frac{1+\sqrt {k}}{k} \Vert x_{n}-y_{n-1} \Vert ^{2}. \end{aligned}$$

By (3.4), (3.9), (3.10), and set $v=w$, we have

3.11 $$\begin{aligned} & \Vert x_{n+1}-w \Vert ^{2}+2 \rho \bigl\Vert (I-P_{Q})Ay_{n} \bigr\Vert ^{2} \\ &\quad\leq \Vert x_{n}-w \Vert ^{2}- \Vert x_{n+1}-x_{n} \Vert ^{2} \\ &\qquad{}-2\rho\bigl\langle x_{n+1}-y_{n},A^{*}(I-P_{Q})Ay_{n}-A^{*}(I-P_{Q})Ay_{n-1} \bigr\rangle \\ &\qquad{}-2\rho\bigl\langle x_{n+1}-y_{n},A^{*}(I-P_{Q})Ay_{n-1} \bigr\rangle -2\rho\bigl\langle y_{n}-w,A^{*}(I-P_{Q})Aw\bigr\rangle \\ &\quad\leq \Vert x_{n}-w \Vert ^{2}- \Vert x_{n+1}-x_{n} \Vert ^{2}+\sqrt{k}\rho \Vert A \Vert ^{2}\cdot \Vert y_{n}-x_{n+1} \Vert ^{2} \\ &\qquad{}+\rho \Vert A \Vert ^{2}\cdot\frac{1+\sqrt{k}}{\sqrt{k}} \Vert y_{n}-x_{n} \Vert ^{2}+\rho \Vert A \Vert ^{2}\cdot\frac{1+\sqrt{k}}{k} \Vert x_{n}-y_{n-1} \Vert ^{2} \\ &\qquad{}+ \Vert x_{n}-x_{n+1} \Vert ^{2}- \Vert x_{n}-y_{n} \Vert ^{2}- \Vert x_{n+1}-y_{n} \Vert ^{2} \\ &\quad= \Vert x_{n}-w \Vert ^{2}-\bigl(1-\sqrt{k}\rho \Vert A \Vert ^{2}\bigr) \Vert x_{n+1}-y_{n} \Vert ^{2}+\rho \Vert A \Vert ^{2}\cdot\frac{1+\sqrt{k}}{k} \Vert x_{n}-y_{n-1} \Vert ^{2} \\ &\qquad{}-\biggl(1-\rho \Vert A \Vert ^{2}\cdot\frac{1+\sqrt{k}}{\sqrt{k}}\biggr) \Vert x_{n}-y_{n} \Vert ^{2}. \end{aligned}$$

By (3.11), we have

3.12 $$\begin{aligned} & \Vert x_{n+1}-w \Vert ^{2}+ \rho \Vert A \Vert ^{2}\cdot\frac{1+\sqrt{k}}{k}\cdot \Vert x_{n+1}-y_{n} \Vert ^{2} \\ &\quad\leq \Vert x_{n+1}-w \Vert ^{2}+\rho \Vert A \Vert ^{2}\cdot\frac{1+\sqrt{k}}{k}\cdot \Vert x_{n+1}-y_{n} \Vert ^{2}+2\rho \bigl\Vert (I-P_{Q})Ay_{n} \bigr\Vert ^{2} \\ &\quad\leq \Vert x_{n}-w \Vert ^{2}-\biggl(1-\sqrt{k}\rho \Vert A \Vert ^{2}-\rho \Vert A \Vert ^{2}\cdot \frac {1+\sqrt{k}}{k}\biggr) \Vert x_{n+1}-y_{n} \Vert ^{2} \\ &\qquad{}+\rho \Vert A \Vert ^{2}\cdot\frac{1+\sqrt{k}}{k} \Vert x_{n}-y_{n-1} \Vert ^{2}-\biggl(1-\rho \Vert A \Vert ^{2}\cdot\frac{1+\sqrt{k}}{\sqrt{k}}\biggr) \Vert x_{n}-y_{n} \Vert ^{2} \\ &\quad\leq \Vert x_{n}-w \Vert ^{2}+\rho \Vert A \Vert ^{2}\cdot\frac{1+\sqrt{k}}{k} \Vert x_{n}-y_{n-1} \Vert ^{2}. \end{aligned}$$

Hence, $\lim_{n\rightarrow \infty} \Vert x_{n}-w \Vert ^{2}+\rho \Vert A \Vert ^{2}\cdot\frac {1+\sqrt{k}}{k}\cdot \Vert x_{n}-y_{n-1} \Vert ^{2}$ exists, and then

3.13 $$ \lim_{n\rightarrow \infty} \Vert y_{n}-x_{n+1} \Vert =\lim_{n\rightarrow \infty } \Vert y_{n}-x_{n} \Vert =\lim_{n\rightarrow \infty} \bigl\Vert (I-P_{Q})Ay_{n} \bigr\Vert =0. $$

Further, this implies that

3.14 $$ \lim_{n\rightarrow \infty} \Vert x_{n}-w \Vert ^{2} =\lim_{n\rightarrow \infty} \biggl( \Vert x_{n}-w \Vert ^{2}+\rho \Vert A \Vert ^{2}\cdot \frac {1+\sqrt{k}}{k}\cdot \Vert x_{n}-y_{n-1} \Vert ^{2} \biggr). $$

So, $\{x_{n}\}_{n\in\mathbb{N}}$ is a bounded sequence, and then there exist $\bar{x}\in C$ and a subsequence $\{x_{n_{k}}\}_{k\in\mathbb {N}}$ of $\{x_{n}\}_{n\in\mathbb{N}}$ such that $x_{n_{k}}\rightharpoonup \bar{x}$. By (3.13), we determine that $y_{n_{k}}\rightharpoonup\bar{x}$ and $Ay_{n_{k}}\rightharpoonup A\bar {x}$. By Lemma 2.1, we know that $A\bar {x}=P_{Q}A\bar{x}$ and $A\bar{x}\in Q$. So, $\bar {x}\in\Omega$. Final, by Opial’s condition, we know that $x_{n}\rightharpoonup\bar{x}$. Therefore, the proof is completed. □

### Remark 3.1

The algorithm in Theorem 3.1 are different from those in the references. For examples, one may refer to [6], Theorem 3.1, [16], Theorem 4.3, [8], Theorem 3.1, [24], Theorem 3.1, Theorem 4.1, and [7], Theorem 3.3.

## Applications

### Convex linear inverse problem

In this section, we consider the following convex linear inverse problem:

$$ (\mathbf{CLIP}) \quad\text{Find } \bar{x}\in C \text{ such that }A\bar{x}=b, $$

where *C* is a nonempty closed convex subset of a real Hilbert space $H_{1}$, *b* is given in a real Hilbert space $H_{2}$, and $A:H_{1}\rightarrow H_{2}$ is a linear and bounded operator.

#### Theorem 4.1

*Let* $H_{1}$ *and* $H_{2}$ *be real Hilbert spaces*, *C* *be a nonempty closed convex subset of* $H_{1}$, $b\in H_{2}$, *and* $A:H_{1}\rightarrow H_{2}$ *be a linear and bounded operator with adjoint operator* $A^{*}$. *Let* Ω *be the solution set of the convex linear inverse problem and assume that* $\Omega\neq\emptyset$. *For* $k>0$, *suppose* *ρ* *satisfies*

$$0< \rho< \min \biggl\{ \frac{\sqrt{k}}{(1+\sqrt{k})\cdot \Vert A \Vert ^{2}},\frac{k}{(k\sqrt{k}+\sqrt{k}+1)\cdot \Vert A \Vert ^{2}} \biggr\} . $$

*Let* $\{x_{n}\}_{n\in\mathbb{N}}$ *be defined by*

4.1 $$ \textstyle\begin{cases} x_{1}\textit{ is chosen arbitrarily in }H_{1},\\ y_{1}=x_{1},\\ x_{n+1}:=P_{C}(x_{n}-\rho A^{*}(Ay_{n}-b)),\\ y_{n+1}:=2x_{n+1}-x_{n},\quad n\in\mathbb{N}. \end{cases} $$

*Then there exists* $\bar{x}\in\Omega$ *such that* $\{x_{n}\}_{n\in \mathbb{N}}$ *converges weakly to* *x̄*.

#### Proof

Let $Q=\{b\}$. Then $P_{Q}(y)=b$ for all $y\in H_{2}$. Hence, we get the conclusion of Theorem 4.1 by using Theorem 3.1. □

### Split equality problem

Let $H_{1}$, $H_{2}$, and $H_{3}$ be real Hilbert spaces. Let *C* and *Q* be nonempty closed convex subsets of $H_{1}$ and $H_{2}$, respectively. Let $A:H_{1}\rightarrow H_{3}$ and $B:H_{2}\rightarrow H_{3}$ be linear and bounded operators with adjoint operators $A^{*}$ and $B^{*}$, respectively. The following problem is the split-equality problem, which was studied by Moudafi [25, 26]:

$$(\mathbf{SEP})\quad \mbox{Find }\bar{x}\in C\mbox{ and }\bar{y}\in Q\mbox{ such that }A\bar {x}=B\bar{y}. $$

Let $\Omega:=\{(x,y)\in C\times Q: Ax=By\}$ be the solution set of problem (**SEP**). Further, we observed that $(x,y)$ is a solution of the split-equality problem if and only if

$$\textstyle\begin{cases} x=P_{C}(x-\rho_{1} A^{*}(Ax-By)),\\ y=P_{Q}(y+\rho_{2} B^{*}(Ax-By)), \end{cases} $$

for all $\rho_{1}>0$ and $\rho_{2}>0$, where $P_{C}$ is the metric projection from $H_{1}$ onto *C*, and $P_{Q}$ is the metric projection from $H_{2}$ onto *Q*, [27].

As mentioned in Moudafi [25], the interest of the split-equality problem covers many situations, for instance in decomposition methods for PDEs, game theory, and modulated radiation therapy (IMRT). For details, see [3, 25, 28]. Besides, we also observed that problem are extended to many generalized problems, like the split-equality fixed point problem [29, 30].

To solve the split-equality problem, Moudafi [26] proposed the alternating CQ algorithm:

$$(\mathbf{ ACQA})\quad \textstyle\begin{cases} x_{1}\text{ and }y_{1}\text{ are chosen arbitrarily in }H_{1}\text{ and }H_{2}, \text{ respectively},\\ x_{n+1}:=P_{C}(x_{n}-\rho_{n} A^{*}(Ax_{n}-By_{n})),\\ y_{n+1}:=P_{Q}(y_{n}+\rho_{n}B^{*}(Ax_{n+1}-By_{n})),\quad n\in\mathbb{N}, \end{cases} $$

where $H_{1}=\mathbb{R}^{N}$, $H_{2}=\mathbb{R}^{M}$, $P_{C}$ is the metric projection from $H_{1}$ onto *C*, and $P_{Q}$ is the metric projection from $H_{2}$ onto *Q*, $\varepsilon>0$, *A* is a $J\times N$ matrix, *B* is a $J\times M$ matrix, $\lambda_{A}$ and $\lambda_{B}$ are the spectral radius of $A^{*}A$ and $B^{*}B$, respectively, and $\{\rho_{n}\}$ is a sequence in $(\varepsilon ,\min\{\frac{1}{\lambda_{A}},\frac{1}{\lambda_{B}}\}-\varepsilon)$.

In 2013, Byrne and Moudafi [31] presented a simultaneous algorithm, which was called the projected Landweber algorithm, to study the split-equality problem:

$$(\mathbf{ PLA})\quad \textstyle\begin{cases} x_{1}\text{ and }y_{1}\text{ are chosen arbitrarily in }H_{1}\text{ and }H_{2}, \text{ respectively},\\ x_{n+1}:=P_{C}(x_{n}-\rho_{n} A^{*}(Ax_{n}-By_{n})),\\ y_{n+1}:=P_{Q}(y_{n}+\rho_{n}B^{*}(Ax_{n}-By_{n})),\quad n\in\mathbb{N}, \end{cases} $$

where $H_{1}=\mathbb{R}^{N}$, $H_{2}=\mathbb{R}^{M}$, $P_{C}$ is the metric projection from $H_{1}$ onto *C*, and $P_{Q}$ is the metric projection from $H_{2}$ onto *Q*, $\varepsilon>0$, *A* is a $J\times N$ matrix, *B* is a $J\times M$ matrix, $\lambda_{A}$ and $\lambda_{B}$ are the spectral radius of $A^{*}A$ and $B^{*}B$, respectively, and $\{\rho_{n}\}$ is a sequence in $(\varepsilon ,\frac{2}{\lambda_{A}+\lambda_{B}})$.

Next, we need the following results to establish our results in the sequel. Let $H_{1}$ and $H_{2}$ be two real Hilbert spaces, $W:=H_{1}\times H_{2}$ with inner product

$$\langle w_{1},w_{2}\rangle=\langle u_{1},u_{2} \rangle_{H_{1}}+\langle v_{1},v_{2} \rangle_{H_{2}} $$

for all $w_{1}=(u_{1},v_{1})$, $w_{2}=(u_{2},v_{2})\in W$. Hence, *W* is a real Hilbert space with norm

$$\Vert z \Vert :=\bigl( \Vert u \Vert _{H_{1}}^{2}+ \Vert v \Vert _{H_{2}}^{2}\bigr)^{1/2},\quad\text{where }z=(u,v)\in W. $$

(For simple, $\langle\cdot,\cdot\rangle_{H_{1}}$ and $\langle\cdot ,\cdot\rangle_{H_{2}}$ are written by $\langle\cdot,\cdot\rangle$.) Further, we know that $\{w_{n}=(u_{n},v_{n})\}\subseteq W=H_{1}\times H_{2}$ converges weakly to $w=(u,v)$ if and only if $\{u_{n}\}$ converges weakly to *u* and $\{v_{n}\}$ converges weakly to *v*. Next, suppose that *C* and *Q* are nonempty closed convex subsets of $H_{1}$ and $H_{2}$, respectively, and set $D=C\times Q\subseteq W$. Then the metric projection $P_{D}(w)=(P_{C}(u),P_{Q}(v))$ for all $z=(u,v)\in W$.

Next, we give a reflected projected Landweber algorithm for the split-equality problem.

#### Theorem 4.2

*Let* $H_{1}$, $H_{2}$, *and* $H_{3}$ *be real Hilbert spaces*. *Let* *C* *and* *Q* *be nonempty closed convex subsets of* $H_{1}$ *and* $H_{2}$, *respectively*. *Let* $A:H_{1}\rightarrow H_{3}$ *and* $B:H_{2}\rightarrow H_{3}$ *be linear and bounded operators with adjoint operators* $A^{*}$ *and* $B^{*}$, *respectively*. *Let* Ω *be the solution set of the split*-*equality problem and assume that* $\Omega\neq\emptyset$. *For* $k>0$, *suppose* *ρ* *satisfies*

$$0< \rho< \min \biggl\{ \frac{\sqrt{k}}{(1+\sqrt{k})\cdot ( \Vert A \Vert ^{2}+ \Vert B \Vert ^{2})},\frac{k}{(k\sqrt{k}+\sqrt{k}+1)\cdot ( \Vert A \Vert ^{2}+ \Vert B \Vert ^{2})} \biggr\} . $$

*Let* $\{x_{n}\}_{n\in\mathbb{N}}$ *and* $\{y_{n}\}_{n\in\mathbb{N}}$ *be defined by*

4.2 $$ \textstyle\begin{cases} x_{1}\textit{ and }y_{1}\textit{ are chosen arbitrarily in }H_{1}\textit{ and }H_{2}, \textit{ respectively},\\ u_{1}=x_{1},\qquad v_{1}=y_{1},\\ x_{n+1}:=P_{C}(x_{n}-\rho A^{*}(Au_{n}-Bv_{n})),\\ y_{n+1}:=P_{Q}(y_{n}+\rho B^{*}(Au_{n}-Bv_{n})),\\ u_{n+1}:=2x_{n+1}-x_{n},\\ v_{n+1}:=2y_{n+1}-y_{n},\quad n\in\mathbb{N}. \end{cases} $$

*Then there exists* $(\bar{x},\bar{y})\in\Omega$ *such that* $\{x_{n}\} _{n\in\mathbb{N}}$ *converges weakly to* *x̄* *and* $\{y_{n}\}_{n\in \mathbb{N}}$ *converges weakly to* *ȳ*.

#### Proof

Let $S=C\times Q$, $G:=[A,\ -B]$, $w=[x\ y]^{T}$, $b=[0\ 0]^{T}$. Then

$$G^{*}G= \begin{bmatrix} A^{*}A & -A^{*}B\\ -B^{*}A & B^{*}B \end{bmatrix} ,\qquad P_{S} \begin{bmatrix} x\\ y \end{bmatrix} = \begin{bmatrix} P_{C}x\\ P_{Q}y \end{bmatrix}. $$

Thus,

$$\begin{bmatrix} x_{n+1}\\ y_{n+1} \end{bmatrix} =P_{S} \left( \begin{bmatrix} x_{n}\\ y_{n} \end{bmatrix} -\rho \begin{bmatrix} A^{*}A & -A^{*}B\\ -B^{*}A & B^{*}B \end{bmatrix} \begin{bmatrix} u_{n}\\ v_{n} \end{bmatrix} \right) $$

and

$$\begin{bmatrix} u_{n+1}\\ v_{n+1} \end{bmatrix} =2 \begin{bmatrix} x_{n+1}\\ y_{n+1} \end{bmatrix} -\begin{bmatrix} x_{n}\\ y_{n} \end{bmatrix} . $$

Therefore, we get the conclusion of Theorem 4.2 by using Theorem 4.1. □

In Theorem 4.2, if we set $H_{2}=H_{3}$ and *B* is the identity mapping on $H_{2}$, then we can obtain a new algorithm and related convergence theorem for the split-feasibility problem.

#### Corollary 4.1

*Let* $H_{1}$ *and* $H_{2}$ *be real Hilbert spaces*. *Let* *C* *and* *Q* *be nonempty closed convex subsets of* $H_{1}$ *and* $H_{2}$, *respectively*. *Let* $A:H_{1}\rightarrow H_{2}$ *be a linear and bounded operator with adjoint operator* $A^{*}$. *Let* Ω *be the solution set of the split*-*feasibility problem* (*SFP*) *and assume that* $\Omega\neq\emptyset $. *For* $k>0$, *suppose* *ρ* *satisfies*

$$0< \rho< \min \biggl\{ \frac{\sqrt{k}}{(1+\sqrt{k})\cdot ( \Vert A \Vert ^{2}+1)},\frac{k}{(k\sqrt{k}+\sqrt{k}+1)\cdot( \Vert A \Vert ^{2}+1)} \biggr\} . $$

*Let* $\{x_{n}\}_{n\in\mathbb{N}}$ *and* $\{y_{n}\}_{n\in\mathbb{N}}$ *be defined by*

4.3 $$ \textstyle\begin{cases} x_{1}\textit{ and }y_{1}\textit{ are chosen arbitrarily in }H_{1}\textit{ and }H_{2}, \textit{ respectively},\\ u_{1}=x_{1},\qquad v_{1}=y_{1},\\ x_{n+1}:=P_{C}(x_{n}-\rho A^{*}(Au_{n}-v_{n})),\\ y_{n+1}:=P_{Q}(y_{n}+\rho(Au_{n}-v_{n})),\\ u_{n+1}:=2x_{n+1}-x_{n},\\ v_{n+1}:=2y_{n+1}-y_{n},\quad n\in\mathbb{N}. \end{cases} $$

*Then there exists* $\bar{x}\in\Omega$ *such that* $\{x_{n}\}_{n\in \mathbb{N}}$ *converges weakly to* *x̄*. *Further*, $\{y_{n}\}_{n\in \mathbb{N}}$ *converges weakly to* *Ax̄*.

#### Remark 4.1

The results in this section are different from those in the references. For example, one may refer to [25], Theorem 2.1.

#### Remark 4.2

From the results in this section, we know that the split-equality problem is a special case of the split-feasibility problem. This is an important contribution in this paper since many researchers thought that the split-feasibility problem is a special case of the split-equality problem.

## Numerical results

All codes were written in R language (version 3.2.4 (2016-03-10)). The R Foundation for Statistical Computing Platform: x86-64-w64-mingw32/x64 (64-bit).

### Example 5.1

Let $H_{1}=H_{2}=\mathbb{R}^{2}$, $C:=\{x\in\mathbb{R}^{2}: \Vert x \Vert \leq1\}$, $Q:=\{x=(u,v)\in\mathbb{R}^{2}: (u-6)^{2}+(v-8)^{2}\leq25\}$, $A=5I_{2}$, where $I_{2}$ is $2\times2$ identity matrix. Then (SFP) has the unique solution $\bar{x}:=(\bar{x}_{1},\bar{x}_{2})\in\mathbb{R}^{2}$. Indeed, $\bar{x}_{1}=0.6$ and $\bar{x}_{2}=0.8$.

We give numerical results for problem (SFP) by using algorithm (PRGA), CQ algorithm, and CQ-like algorithm. Let $\varepsilon>0$ and the algorithm stop if $\Vert x_{n}-\bar{x} \Vert <\varepsilon$.

In Tables 1 and 2, we set $x_{1}=(10,10)^{T}$, $\rho_{n}=\rho=0.06$ for all $n\in\mathbb{N}$. From Table 1, we see that the proposed algorithm in Theorem 3.1 reaches the required errors faster than the CQ algorithm and CQ-like algorithms with $w_{n}=1$ (resp. $w_{n}=1.9$). From Tables 2 and 1, we see that the proposed algorithm in Theorem 3.1 only need 6,402,868 iteration number and 150.65 seconds to reach the required error $\varepsilon=10^{-7}$, but the other algorithms could not reach the required error.

**Table 1:** **Numerical results for Example** **5.1** **(** $\pmb{x_{1}=(10,10)^{T}}$ **,** $\pmb{\rho_{n}=\rho=0.06}$ **for all** $\pmb{n\in\mathbb {N}}$ **)**

***ε***	**CPU(s)**	**Iteration**	**Approximate solution**	**CPU(s)**	**Iteration**	**Approximate solution**
	CQ algorithm			PRGA		
10^−3^	-	2	(0.5994553, 0.8004082)	0.01	314	(0.6006783, 0.7994908)
10^−4^	375.25	1,630,698	(0.5999200, 0.8000600)	0.03	1334	(0.5999466, 0.8000400)
	CQ-like algorithm ($w_{n}=1$)			CQ-like algorithm ($w_{n}=1.9$)		
10^−3^	6.47	249,918	(0.6007997, 0.7993996)	3.41	131,247	(0.6007997, 0.7993996)
10^−4^	652.44	24,999,920	(0.6000800, 0.7999400)	342.80	13,157,560	(0.6000800, 0.7999400)

**Table 2:** **Numerical results for Example** **5.1**

	$\mathbf{PRGA}\ \boldsymbol {[x_{1}=(10,10)^{T}, \rho=0.06]}$		
***ε***	**CPU(s)**	**Iteration**	**Approximate solution**
10^−3^	0.01	314	(0.6006783, 0.7994908)
10^−4^	0.03	1334	(0.5999466, 0.8000400)
10^−5^	0.10	3741	(0.6000052, 0.7999961)
10^−6^	14.67	650,838	(0.5999999, 0.8000001)
10^−7^	150.65	6,402,868	(0.6000001, 0.8000000)

In Tables 3 and 4, we set $x_{1}=(1,1)^{T}$, $\rho_{n}=\rho=0.06$ for all $n\in\mathbb{N}$. From Table 3, we see that the proposed algorithm in Theorem 3.1 reaches the required errors faster than the CQ algorithm. From Tables 4 and 3, we see that the proposed algorithm in Theorem 3.1 only needs 1,058,254 iterations and 374.21 seconds to reach the required error $\varepsilon=10^{-7}$, but the CQ algorithm could not reach the required error.

**Table 3:** **Numerical results for Example** **5.1** **(** $\pmb{x_{1}=(1,1)^{T}}$ **,** $\pmb{\rho_{n}=\rho=0.06}$ **for all** $\pmb{n\in\mathbb{N}}$ **)**

	**CQ algorithm**			**PRGA**		
***ε***	**CPU(s)**	**Iteration**	**Approximate solution**	**CPU(s)**	**Iteration**	**Approximate solution**
10^−3^	3.89	166,658	(0.6007997, 0.7993996)	-	375	(0.5993223, 0.8005078)
10^−4^	374.21	16,666,660	(0.6000800, 0.7999400)	0.15	7086	(0.5999597, 0.8000302)

**Table 4:** **Numerical results for Example** **5.1**

	$\mathbf{PRGA}\ \boldsymbol {[x_{1}=(1,1)^{T}, \rho=0.06]}$		
***ε***	**CPU(s)**	**Iteration**	**Approximate solution**
10^−3^	−	375	(0.5993223, 0.8005078)
10^−4^	0.16	7086	(0.5999597, 0.8000302)
10^−5^	0.22	9493	(0.5999947, 0.8000040)
10^−6^	1.00	44,211	(0.6000002, 0.7999999)
10^−7^	24.63	1,058,254	(0.6, 0.8)

## Conclusions

In this paper, we study the split-feasibility problem in Hilbert spaces by using the projected reflected gradient algorithm. From the proposed numerical results, we know the projected reflected gradient algorithm is useful and faster than the CQ algorithm and CQ-like algorithms under suitable conditions. As applications, we study the convex linear inverse problem and the split-equality problem in Hilbert spaces. Here, we give an important connection between the linear inverse problem and the split-equality problem. Hence, many modified projected Landweber algorithms for the split-equality problem will be presented by using the related algorithms for the linear inverse problem.
